# Pan-Cancer Detection Through DNA Methylation Profiling Using Enzymatic Conversion Library Preparation with Targeted Sequencing

**DOI:** 10.3390/ijms262010165

**Published:** 2025-10-19

**Authors:** Alvida Qvick, Emma Adolfsson, Lina Tornéus, Carl Mårten Lindqvist, Jessica Carlsson, Bianca Stenmark, Christina Karlsson, Gisela Helenius

**Affiliations:** 1 Clinical Research Center, Faculty of Medicine and Health, Örebro University, SE-701 85 Örebro, Sweden; 2 Department of Obstetrics and Gynecology, Faculty of Medicine and Health, Örebro University, SE-701 85 Örebro, Sweden; emma.adolfsson@regionorebrolan.se; 3 Department of Laboratory Medicine, Örebro University Hospital, SE-701 85 Örebro, Sweden; 4 Department of Urology, Faculty of Medicine and Health, Örebro University, SE-701 85 Örebro, Sweden; 5 School of Health Sciences, Örebro University, SE-701 85 Örebro, Sweden; 6 ATMP Center, Skåne University Hospital, SE-222 42 Lund, Sweden; gisela.helenius@skane.se; 7 Faculty of Medicine and Health, Örebro University, SE-701 85 Örebro, Sweden

**Keywords:** cfDNA, methylation, pan-cancer, epigenetics, next-generation sequencing, liquid biopsy

## Abstract

We investigated differences in circulating cell-free DNA (cfDNA) methylation between patients with cancer and those presenting with severe, nonspecific symptoms. Plasma cfDNA from 229 patients was analyzed, of whom 37 were diagnosed with a wide spectrum of cancer types within 12 months. Samples underwent enzymatic conversion, library preparation, and enrichment using the NEBNext workflow and Twist pan-cancer methylation panel, followed by sequencing. Methylation analysis was performed with nf-core/methylseq. Differentially methylated regions (DMRs) were identified with DMRichR. Machine learning with cross-validation was used to classify cancer and controls. The classifier was applied to an external validation set of 144 controls previously unseen by the model. Cancer samples showed higher overall CpG methylation than controls (1.82% vs. 1.34%, *p* < 0.001). A total of 162 DMRs were detected, 95.7% being hypermethylated in cancer. Machine learning identified 20 key DMRs for classification between cancer and controls. The final model achieved an AUC of 0.88 (83.8% sensitivity, 83.8% specificity), while mean cross-validation performance reached an AUC of 0.73 (57.1% sensitivity, 77.5% specificity). The specificity of the classifier on unseen control samples was 79.2%. Distinct methylation differences and DMR-based classification support cfDNA methylation as a robust biomarker for cancer detection in patients with confounding conditions.

## 1. Introduction

Liquid biopsy is increasingly recognized for its many applications in cancer management, including early detection, diagnostics, surveillance, and prognosis [1]. The most researched biomarker in liquid biopsies is circulating cell-free DNA (cfDNA), which is released from cells through apoptosis, necrosis, and active secretion. Due to their high turnover rate, tumor cells release more DNA, referred to as circulating tumor DNA (ctDNA), compared to normal cells [2]. Both cfDNA and ctDNA carry complete genetic and epigenetic information from their cell of origin, including mutations, larger genetic abnormalities such as copy number variants, and methylation patterns [3].

Epigenetics refer to heritable changes in gene expression that occur without alterations to the underlying DNA sequence. Key epigenetic mechanisms include DNA methylation, histone modifications, and chromatin remodeling, all of which regulate gene activity and maintain cellular identity. In malignancy, aberrant methylation patterns disrupt normal gene regulation, contributing to tumor initiation and progression. Since these epigenetic changes occur early in cancer development and are relatively stable, they are particularly attractive as biomarkers for early detection [4].

Mutational profiling of cancer via liquid biopsy has been the most investigated biomarker approach and was the first to receive FDA approval for detecting resistance mutations in lung cancer [5]. However, point mutations in ctDNA are rare and are often found at low allele frequencies, which decrease further as the cancer evolves and tumor heterogeneity increases. As a result, these analyses are less well-suited for reliable cancer detection [6,7]. On the other hand, epigenetic patterns such as methylation are established early in cancer development, and analyzing multiple loci enhances the likelihood of accurate detection [8,9,10]. The most common methylation pattern in mammals involves the addition of a methyl group to the 5-carbon position of cytosines, usually in the context of CpG dinucleotides [11]. In cancer, methylation alterations can be categorized into focal hypermethylation and global hypomethylation [12,13,14]. Methylation patterns include differentially methylated regions (DMRs), which are defined as sets of CpGs in close proximity. Due to the harsh chemical treatment usually performed before methylation analysis, methylation-based cfDNA analyses have not been feasible, and few studies have investigated their use in a pan-cancer context [15,16].

In this study, we employed targeted next-generation sequencing (NGS)-based analysis combined with a novel cytosine conversion method, using an enzymatic methodology that is less damaging to DNA than conventional bisulfite treatment and consequently allows for low-input DNA. Using this method, we investigated methylation patterns in cfDNA as a diagnostic aid in a pan-cancer setting, focusing on patients presenting with severe, nonspecific symptoms suggestive of cancer.

The aim of the study was to investigate the feasibility of enzymatic conversion methylation sequencing for liquid biopsy. The aim was also to investigate the discriminative power of a targeted methylation-based NGS panel for cancer detection in a clinical pan-cancer setting.

## 2. Results

### 2.1. Patient Characteristics

The complete cohort consisted of 229 patients with an even sex distribution and a median age of 72 years (Table 1). The cohort was divided into three groups: patients with nonmalignant conditions (*n* = 183), patients with a cancer diagnosis within 12 months (*n* = 37) and patients that received a cancer diagnosis later than 12 months from start of investigation (*n* = 9). The cancer group included a slightly higher proportion of females (not statistically significant (n.s.)) and was significantly older than the nonmalignant control group (*p* = 0.001, Table 1). The sub-cohort used for DMR analysis consisted of all cancer cases with age- and sex-matched controls from the control group; hence the baseline characteristics are not reported separately for these controls. The clinical characteristics of the patients belonging to the Late cancer group can be seen in Supplemental Table S2.

The cancer group primarily consisted of carcinomas (67.6%), with adenocarcinomas being the largest subtype (60%), originating from various anatomical sites. Haematolymphoid cancers accounted for 24.3% of the cancer cases. Among patients with solid tumors, 37.9% had metastatic disease at the time of diagnosis. Details are provided in Supplemental Table S1.

The control group encompassed a broad spectrum of diagnoses (Table 1), with further details available in Supplemental Table S1. The largest subgroup consisted of patients diagnosed with various autoimmune diseases (13.5%), of which polymyalgia rheumatica accounted for the majority (50.0%). Infectious diseases accounted for another significant subgroup (11.5%), predominantly bacterial in origin (80.0%), with further details available in Supplemental Table S1.

### 2.2. Feasibility

**Figure 1:**
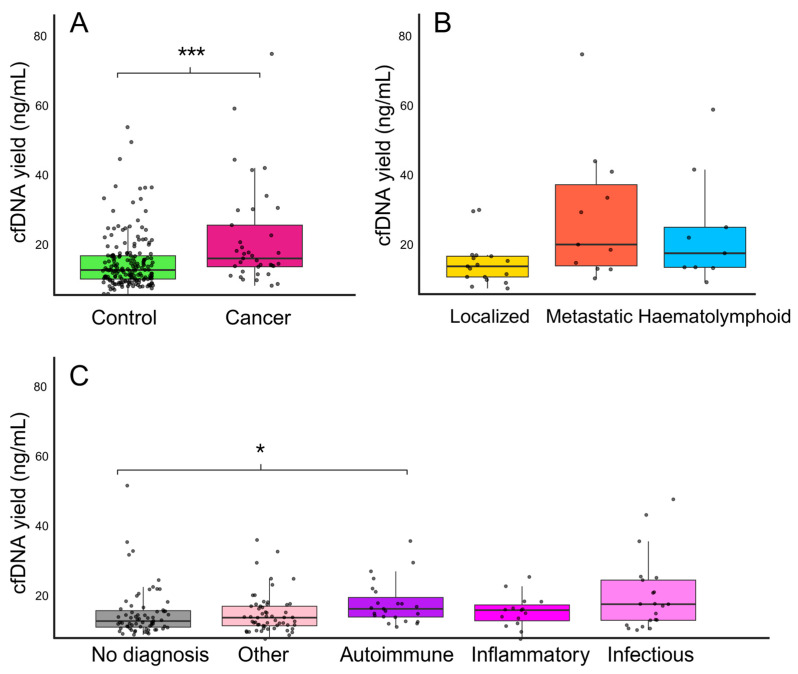
cfDNA yield after extraction. Each dot represents one sample. (**A**) Comparison of cfDNA yield between controls (*n* = 183) and cancer patients (*n* = 37). Cancer patients exhibited significantly higher levels of cfDNA (*p* = 0.0008) compared to controls. (**B**) cfDNA levels in cancer patients categorized by disease status: localized disease (*n* = 17), metastatic disease (*n* = 11), and haematolymphoid origin (*n* = 9). Patients with metastatic disease and haematolymphoid malignancies had higher cfDNA yield than those with localized disease, but the difference did not reach statistical significance. (**C**) cfDNA levels in control patients categorized by diagnosis: patients with autoimmune disease (*n* = 23) had significantly higher cfDNA yield compared to patients with no diagnosis (*n* = 67) (*p* = 0.03). Significance levels: * *p* < 0.05, *** *p* < 0.001. Created in R and modified in BioRender, Adolfsson, E. (2025) https://BioRender.com/6jf5jo7 (accessed on 15 October 2025).

The cfDNA extraction yield had a median concentration of 8.3 ng/mL plasma (IQR: 5.0–13.1 ng/mL, range: 0.0–76.7 ng/mL), with two samples from the control group and one sample from Later cancer having undetectable concentrations. Compared with controls, cancer patients had a significantly higher median cfDNA concentration of 11.4 ng/mL (IQR: 8.7–22.0 ng/mL, range: 2.6–76.7 ng/mL), whereas controls had a median of 7.7 ng/mL (IQR: 4.8–12.2 ng/mL, range: 0.0–53.3 ng/mL, *p* = 0.0008) (see Figure 1A). The Later cancer group had a median cfDNA concentration of 8.8 ng/mL (range 0–48.6 ng/mL).

When cancer cases were stratified by disease type, metastatic (median 16.5 ng/mL, IQR: 9.8–35.5 ng/mL, range: 5.8–76.7 ng/mL) and haematolymphoid malignancies (median 13.8 ng/mL, IQR: 9.3–22.0 ng/mL, range 4.6–59.3 ng/mL) showed higher median cfDNA concentrations and greater variability compared to localized cases (median 9.6 ng/mL, IQR: 6.3–12.8 ng/mL, range 2.6–27.5 ng/mL), although these differences did not reach statistical significance (*p* = 0.06) (see Figure 1B).

When controls were stratified by diagnosis, autoimmune cases (median 10.5 ng/mL, IQR 7.6–14.4 ng/mL, range 4.2–34.0 ng/mL) exhibited statistically significant higher cfDNA concentration compared to controls with no diagnosis (median 6.2, IQR 4.1–9.9 ng/mL, range 1.4–53.3 ng/mL, *p*.adj = 0.03). Infectious (median 12.1 mg/mL) and Inflammatory conditions (median 10.0 ng/mL) also displayed higher cfDNA levels, although these differences did not reach statistical significance. The heterogenous “Other” control group had a low cfDNA concentration (median 7.4 ng/mL) (see Figure 1C).

No significant correlation was observed between BMI and cfDNA concentration in the overall cohort (ρ = 0.13, *p* = 0.06). In controls, a weak positive correlation was detected (ρ = 0.19, *p* = 0.01), while no correlations were found in cancer or later cancer. Age was positively correlated with cfDNA concentration in the full cohort (ρ = 0.28, *p* < 0.00001), primarily driven by controls (ρ = 0.26, *p* = 0.00045). No sex differences were observed overall (*p* = 0.68; men: median 8.3 ng/mL, IQR 7.4; women: median 9.1 ng/mL, IQR 7.8). Within the cancer group, women had higher concentrations than men (13.5 ng/mL, IQR 18.9 vs. 8.9 ng/mL, IQR 7.0; *p* = 0.045). No differences were observed in controls or Later cancer.

All samples with measurable cfDNA concentrations were processed for library preparation, with total input amounts ranging from 2.6 to 146.5 ng and a median input of 16.9 ng. All those samples successfully generated high-quality libraries and were sequenced, generating a median of 12.6 M reads (IQR: 10.1–15.9 M, range: 5.1–30.0 M reads). The median coverage was 659× (IQR: 511–844×, range: 208–1534×), with an on-target rate of 73.6% (IQR: 70.1–76.9%, range: 60.0–83.0%). Cancer samples had significantly higher median coverage than controls (median 857× vs. 652×, *p* = 0.006); however, this difference was no longer significant after adjusting for input cfDNA (*p* = 0.75). The median GC content was 33% (range: 31–37%) and showed a moderate positive correlation with input cfDNA amount, even after adjusting for cancer status (β: 0.63 CI: 0.024–0.033, *p* < 0.001). Input amount was positively correlated with coverage (*rho* = 0.76, *p* < 0.001) and negatively correlated with duplication rate (*rho* = −0.74, *p* < 0.001).

### 2.3. Overall CpG Methylation

To assess the conversion rate, commercial controls containing Lambda and pUC19 DNA were analyzed. All sequenced control libraries met the expected methylation levels, with unmethylated Lambda showing 0.25–0.26% methylation and methylated pUC19 showing 95.82–96.02% methylation.

**Figure 2:**
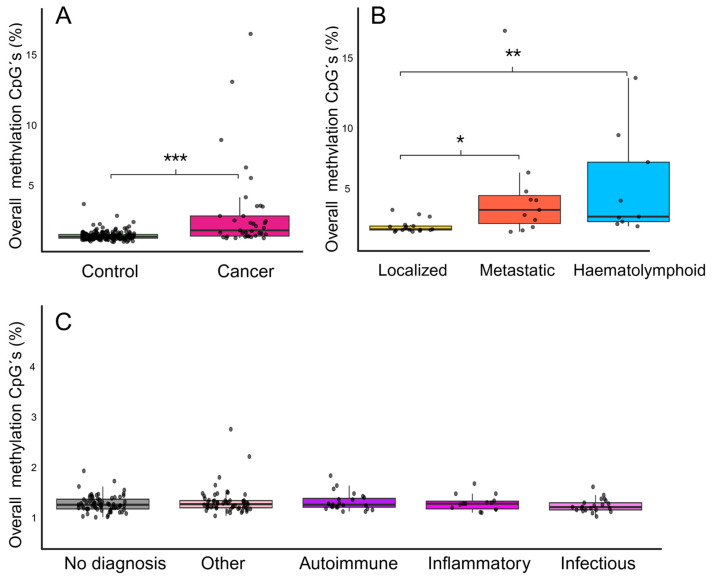
Panel-wide CpG methylation levels. Each dot represents one sample. (**A**) Comparison of overall CpG methylation levels between controls (*n* = 181) and cancer patients (*n* = 37). Cancer patients exhibited significantly increased methylation levels (*p* < 0.001) compared to controls. (**B**) Overall CpG methylation levels in cancer patients categorized by disease status: localized disease (*n* = 17), metastatic disease (*n* = 11), and haematolymphoid malignancies (*n* = 9). Patients with metastatic disease had significantly higher methylation levels than those with localized disease (*p* = 0.027), as did patients with haematolymphoid malignancies (*p* = 0.002). (**C**). Overall CpG methylation levels in controls. No statistically significant differences were observed between the control groups. Significance levels: * *p* < 0.05, ** *p* < 0.01, *** *p* < 0.001. Created in R and modified in BioRender. Adolfsson, E. (2025) https://BioRender.com/uiyraoj (accessed on 15 October 2025).

Compared to controls, cancer samples presented statistically significant higher overall CpG methylation across the entire panel, with a median of 1.82% (IQR 1.38–2.93%, range: 1.21–16.94%), versus 1.34% (IQR 1.22–1.52%, range: 0.93–6.63, *p* = 0.00252) (see Figure 2A). This difference remained significant after adjusting for sample age and input amount (OR: 2.37, 95% CI: 1.36–4.12, *p* = 0.002).

When cancer cases were stratified by metastatic status at the time of diagnosis, patients with metastases had significantly higher overall CpG methylation (median 2.93%, IQR 1.86–4.05%, range: 1.22–16.94%, *n* = 11) compared to those with localized disease (median 1.15%, IQR 1.36–1.67%, range: 1.22–2.93%, *n* = 17, *p* = 0.027). Cancers of haematolymphoid origin displayed methylation levels comparable to those of patients with metastatic disease (median 2.40%, IQR 2.01–6.66%, range: 1.65–13.25%, *n* = 9, *p* = 1.0) but had significantly higher overall methylation levels than patients with localized disease (*p* = 0.006); see Figure 2B.

When controls were stratified by diagnosis, there was no statistically significant differences in overall CpG methylation levels between the groups (*p* = 0.49). Median methylation levels were low and comparable between all groups: Autoimmune—median 1.35%, IQR 1.26–1.55%, range: 1.11–2.31%, *n* = 23; Infectious—median 1.26%, IQR 1.17–1.41%, range: 0.96–1.94%, *n* = 21; Inflammatory—median 1.38%, IQR: 1.21–2.05%, range 1.08–2.05%, *n* = 15; Other—median 1.37%, IQR 1.24–1.48%, range 0.98–3.85%, *n* = 57; and No diagnosis—median 1.35%, IQR 1.20–1.54%, range: 0.93–2.47%, *n* = 67) (see Figure 2C).

The Later cancers presented with median overall CpG methylation of 1.35% (range 1.09–6.63%). One of the cases had nearly twice the overall CpG methylation level (6.63%) compared to the highest control sample (3.89%). This patient had primary biliary cholangitis at the time of liquid biopsy, a condition associated with a significantly increased risk of hepatocellular cancer compared to matched individuals from the general population [17]. This patient was later diagnosed with metastatic lung cancer.

No significant correlation was found between BMI and methylation in the overall cohort (ρ = −0.02, *p* = 0.82). Age was positively correlated with methylation (ρ = 0.17, *p* < 0.01), explained by controls (ρ = 0.15, *p* = 0.047), with no significant findings in cancer or later cancer. No sex differences were seen overall (*p* = 0.40; men: median 1.4, IQR 0.4; women: median 1.4, IQR 0.4). In the cancer group, methylation was higher in women compared with men (2.5, IQR 2.2 vs. 1.6, IQR 0.8; *p* = 0.015). No sex-related differences were observed in controls or later cancer.

### 2.4. Detection of DMRs

**Figure 3:**
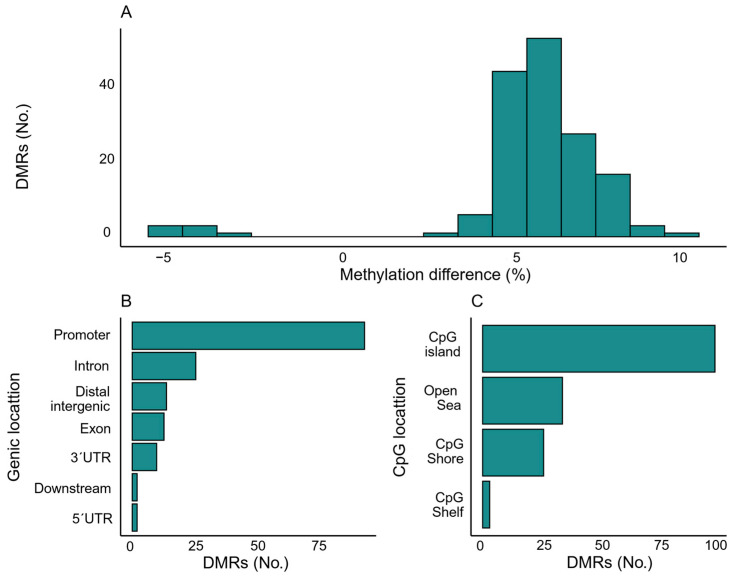
Distribution and localization of DMRs identified using DMRichR. (**A**) Number of DMRs categorized by the magnitude of methylation difference between cancer patients and controls. (**B**) Genic localization of DMRs, showing their distribution across various regions. (**C**) CpG localizations of DMRs, depicting their distribution within different CpG contexts.

For detection of DMRs, 37 cancer cases (diagnosed within 12 months) and sex- and age-matched controls (*n* = 37) were selected. A total of 162 significant DMRs were identified between cancer patients and controls (Supplemental Table S3). These DMRs had a median length of 431 bp (IQR 203–798 bp, range: 79–2431 bp) and contained a median of 24 CpGs (IQR 8–40 bp, range: 5–133 CpGs). The vast majority of DMRs were hypermethylated in cancer, with 155 regions (95.7%) showing hypermethylation, while only 7 regions (4.3%) were hypomethylated. The mean difference in methylation levels across the DMRs was 6% (IQR 5–7%, range: −4–10%) (Figure 3A). Regarding gene locations, 58.6% of the DMRs (*n* = 95) were located in promoter regions, while 31.5% (*n* = 51) were located within gene bodies (including exons, introns, 5′ UTR and 3′ UTR, Figure 3B). When categorized by the CpG context, the majority of DMRs were found within CpG islands (*n* = 103, 63.6%, Figure 3C). Due to the relatively small number of cases within each tumor type, we were unable to perform reliable DMR analyses stratified by cancer subtype; therefore, all DMR results are presented at the global level (cancer vs. control).

Enrichment analysis compared the significant hypermethylated DMRs against background regions across different CpG contexts, including CpG islands, shores, shelves, and the open sea, as well as within gene regions annotated as promotors, untranslated regions, exons, introns, and downstream or intergenic regions. The significantly hypermethylated DMRs showed enrichment in the CpG shores compared to background regions (OR = 2.17, 95% CI = 1.53–3.08, *p* < 0.0001). The limited number of hypomethylated DMRs precluded meaningful enrichment analysis.

**Figure 4:**
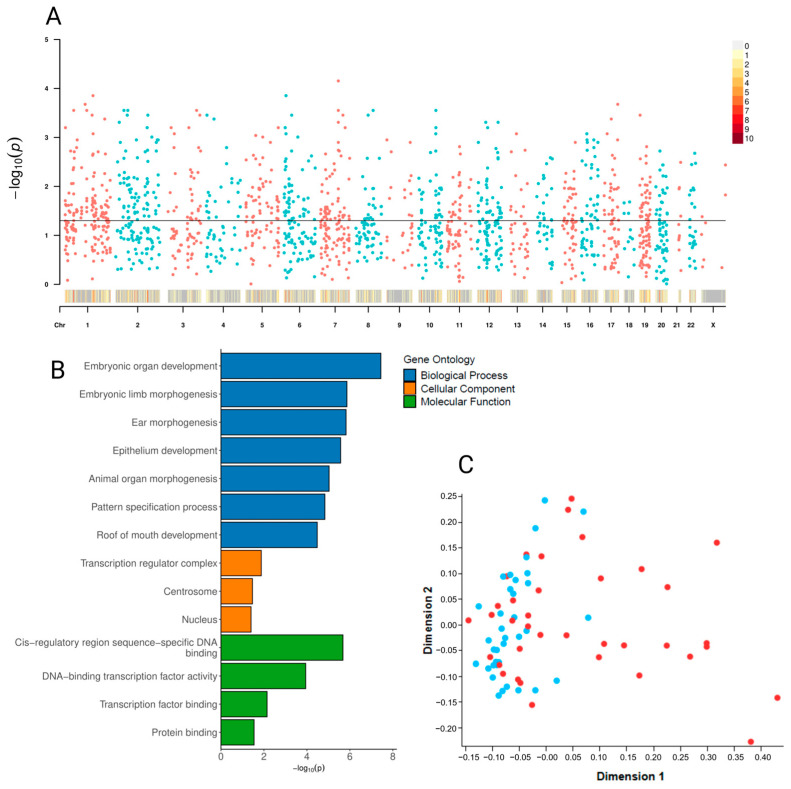
Biological properties of Differentially Methylated Regions (DMRs). (**A**) Manhattan genomic coordinate dot plots of DMRs. Each dot represents a DMR. Colors indicate chromosomal belonging (red for odd numbers and X, blue for even numbers) with those above the black line indicating regions significantly different between cancer and control samples, and those below representing background regions. The bottom scale displays the density of DMRs across the genome. (**B**) Gene ontology analysis using rGREAT. Top enriched terms are shown for biological processes, cellular components, and molecular functions. (**C**) Multidimensional scaling plot showing moderate separation between cancer patients (red) and controls (blue), with each dot representing one individual sample.

The DMRs were dispersed across the genome (see Figure 4A). Ontology analysis revealed that these regions were associated with biological processes related to transcription regulation, organ development, and morphogenesis. Cellular component localization was predominantly associated with the transcription regulator complex, centrosome and nuclear origin, while molecular functions were primarily associated with transcription and sequence-specific DNA binding (see Figure 4B). A multidimensional scaling plot further revealed moderate separation between cancer and control groups (see Figure 4C).

Clustering the samples based on the significant DMRs showed moderate separation between cancers and controls (Supplemental Figure S1).

### 2.5. Machine Learning and Model Building

**Figure 5:**
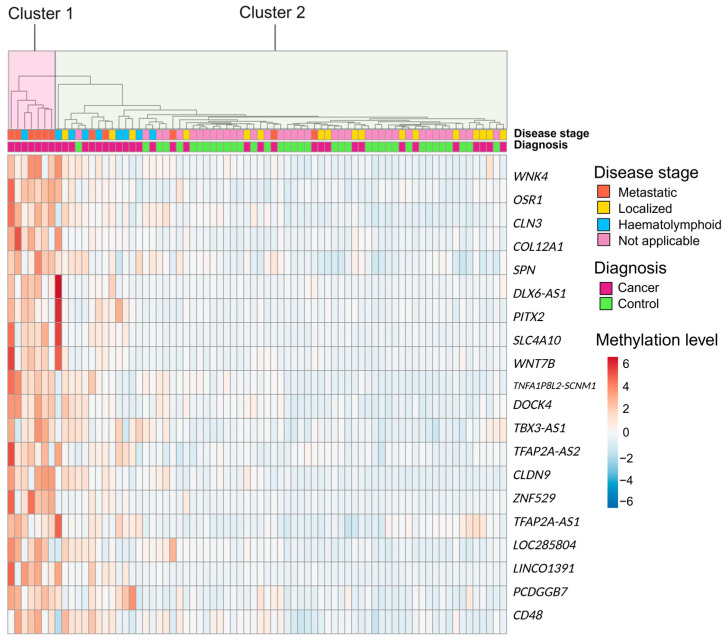
Heatmap of the 20 DMRs selected for model construction. Unsupervised hierarchical clustering of all samples based on methylation levels at the selected DMRs. Samples are annotated by diagnosis (cancer vs. control), with cases further stratified by disease stage (localized, metastatic or haematolymphoid). All control samples are labeled as “Not applicable” for disease stage. Created in R and modified in BioRender. Adolfsson, E. (2025) https://BioRender.com/o1kopgh (accessed on 15 October 2025).

Using the sigFeature machine learning approach, 20 DMRs were identified (see Supplemental Table S3 for feature data and Supplemental Figure S2 for an exemplary plot) and subsequently used as input features for constructing a classification model to distinguish cancer patients from controls. The top 20 DMRs’ discriminatory capacity is illustrated in a hierarchical clustering heatmap (see Figure 5). Visual inspection of the dendrogram clearly separates the samples into two biologically meaningful clusters, one large mixed cluster (cancer = 30, controls = 37) and one smaller cluster with only cancer samples (*n* = 7). All localized cancers were clustered in the mixed large cluster, while the cancer cluster predominately contained metastatic cancers. This cluster is characterized by high methylation levels in the selected DMRs.

**Figure 6:**
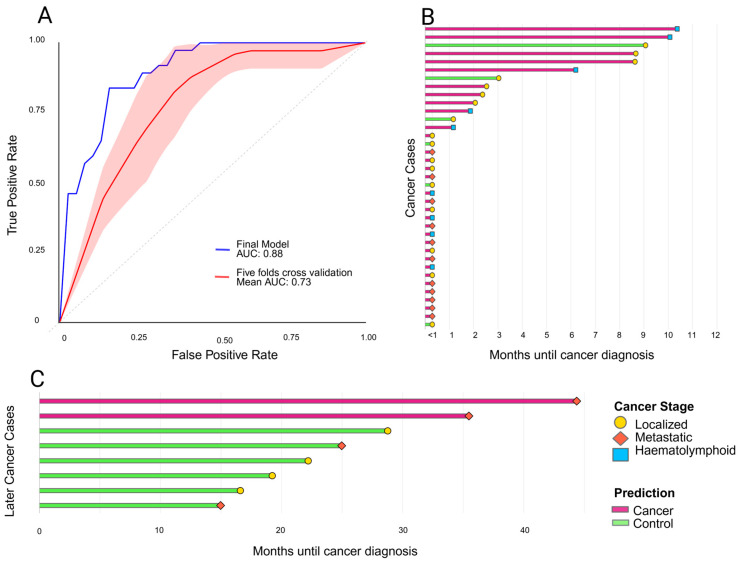
Predictive performance and temporal distribution of cancer diagnoses (**A**) Receiver operating characteristics (ROC) curves illustrating the performance of the final support vector machine (SVM) model (AUC = 0.88) compared with five-fold cross-validation (mean AUC = 0.73). The shaded area represents the variability across folds, reflecting differences in model performance on independent data folds. (**B**) Horizontal bar plot of cancer cases by months until cancer diagnosis. Bars represent predicted class by model, and stage-specific markers indicate disease stage. (**C**) Horizontal bar plot of Later cancers by months until diagnosis. Bars represent predicted class by model, with stage-specific markers for localized and metastatic disease. Created in R, modified in BioRender. Adolfsson, E. (2025) https://BioRender.com/9aoje6t (accessed on 15 October 2025).

The final model achieved a sensitivity of 83.8% and a specificity of 83.8% (AUC: 0.88), indicating a moderate discriminative ability to correctly identify cancer cases from methylation patterns. To further assess robustness, a five-fold cross-validation was performed; across the folds the mean sensitivity and specificity were 57.1% and 77.5%, respectively, with a mean AUC of 0.73, suggesting that while the model performs well on the training split, its performance decreases under cross-validation, reflecting variability across subsets of the data (see Figure 6A). Applied to the training dataset, the model accurately classified 31 of 37 cancers as cancers, where all metastatic and haematolymphoid cancer cases were correctly predicted (see Figure 6B). Investigating the different control groups further, all groups had a specificity higher than 80% except for the autoimmune disease group (57.1%) (see Table 2).

The model was then applied to the remaining controls in the cohort receiving an overall specificity of 79.2%. The model had a relatively even performance among the different groups of diagnoses, with autoimmune diseases having the highest specificity (87.5%) and the no diagnosis group having the lowest (75.0%) (see Table 3).

Among the Later cancers, eight had measurable cfDNA concentration and were used to test the classifiers’ ability to detect cancer in patients who received their cancer diagnosis after 12 months from start of clinical investigation (see Table 4). Interestingly, the model correctly classified the two metastatic cases with the longest time until cancer diagnosis (see Figure 6C).

## 3. Discussion

In this study, we employed an NGS-based targeted methylation panel for pan-cancer analysis to explore the feasibility of noninvasive cancer detection using plasma cfDNA. We successfully demonstrated that this approach is applicable in a low-input setting, showing robust performance even when only limited cfDNA amounts were available. The analysis revealed distinct methylation patterns that distinguished cancer patients from individuals with severe, nonspecific symptoms, both at a global methylation level and across specific DMRs throughout the genome. A classification model based on the 20 most informative DMRs achieved a sensitivity and specificity of 83.8%, and when applied to the remaining controls, the classifier correctly predicted 79.2% of cases.

In the past, methylation analysis traditionally relied on either affinity-based enrichment methods (~100 bp resolution) or sequencing with single-base resolution [18,19]. Additionally, sequencing used to depend on bisulfite conversion of unmethylated cytosines to uracil, which substantially damages DNA and necessitates large input amounts [20]. This made them poorly suited for cfDNA, particularly in patients with compromised health and limited sample availability. The enzymatic conversion method used in our study, in contrast, is highly compatible with low-input cfDNA and enabled successful library preparation from as little as 2.6 ng of cfDNA. While similar enzymatic approaches have been evaluated previously [21,22], they have not been specifically tested in cfDNA. Our results revealed a strong correlation between input amount, coverage, and duplication rate, yet high-quality libraries were consistently obtained. This indicates that strict input thresholds are unnecessary—a crucial advantage since patients with nonmalignant diseases often have lower cfDNA concentrations [23]. Excessive input requirements could otherwise exclude a substantial number of patients and thereby limit clinical applicability.

All study participants were recruited at their first visit within the cancer pathway for severe, nonspecific symptoms, when their cancer status was unknown. Consequently, our cohort was imbalanced, comprising 37 cancer cases, 183 non-healthy controls, and nine individuals who later developed cancer. This imbalance mirrors the clinical reality of the pathway, where only a minority of patients ultimately receive a cancer diagnosis. The cancer detection rate in our cohort (~17%) aligns with previously reported data for this diagnostic pathway [24,25].

Despite the heterogeneity in our cohort, we observed differences in overall methylation levels between cancer patients and controls, with cancer patients exhibiting higher methylation levels. Further analysis revealed that patients with metastatic disease displayed statistically significant higher methylation levels than those with localized tumors. This is likely due to a higher proportion of ctDNA relative to cfDNA in the bloodstream, consistent with established findings that ctDNA concentrations increase with advancing disease stage [7,26,27]. Similarly, haematolymphoid cancers—which have direct access to the circulation—showed elevated methylation levels comparable to metastatic cancers [28]. Since a large fraction of cfDNA (>70%) originates from leukocytes [29], such cancers may be more readily detectable in plasma. These findings raise an important question: do methylation-based biomarkers primarily reflect tumor-specific processes, or are they also influenced by systemic effects associated with advanced disease? This remains a critical challenge for cfDNA-based biomarker interpretation.

The classifier demonstrated moderate performance on the training data (mean sensitivity 83.8%, specificity 83.8%, AUC 0.88) but decreased accuracy during cross-validation (mean sensitivity 57.1%, specificity 77.5%, AUC 0.73), indicating a potential risk of overfitting. This discrepancy likely stems from the limited cohort size and sample heterogeneity. However, the specificity remained relatively stable (79.2%) when applied to the remaining controls (*n* = 144), suggesting some generalizability with respect to specificity. Importantly, the classifier showed similar performance across all control subgroups, indicating consistent behavior regardless of underlying nonmalignant conditions.

The results of our methylation-based classifier are consistent with previous studies demonstrating the potential of methylation biomarkers for cancer detection across multiple tumor types [30,31,32,33]. In pan-cancer settings, the GRAIL test achieved an overall sensitivity of 76.4% and specificity of 99.3% for 12 common cancers, though sensitivity decreased to 54.9% when expanded to 50 tumor types [16]. Other large studies, such as the Thunder trial (NCT04820868), reported a sensitivity of 69% and specificity of 99% [34], while the PanSeer test demonstrated 88% sensitivity and 96% specificity when comparing cancer cases to healthy control and was able to detect cancers up to four years before clinical diagnosis [15]. These results collectively illustrate the promise of methylation-based assays for early cancer detection and support ongoing clinical trials (NCT03934866, NCT04241796, NCT03085888).

One of the most prominent DMRs in the final model was located near the *WNT7B* gene, which was hypermethylated in cancer patients (6% difference compared to controls). *WNT7B* encodes a protein involved in Wnt signaling and has been implicated in several cancer types, including hepatocellular carcinoma [35], colorectal cancer [36], and prostate cancer [37], as well as in metastasis of breast and pancreatic adenocarcinomas [38]. Aberrant methylation of this locus has also been linked to gastric cancer [39] and was recently evaluated as a pan-cancer biomarker [40]. These findings underscore the biological relevance of the DMRs identified and support their potential significance in both general and cancer-type-specific contexts.

By including patients with severe, nonspecific symptoms as controls, our study extends previous work by evaluating a clinically relevant and heterogeneous cohort. While this heterogeneity may have reduced specificity compared to studies using healthy controls, it enhances the translational value of our findings. It demonstrates that methylation-based biomarkers retain discriminatory power even amid substantial biological variability and background noise from other severe, nonmalignant conditions. The inclusion of clinically relevant controls is therefore a major strength of this study.

The inclusion criteria for cancer patients, diagnosed within 12 months of sampling, were designed to associate symptoms directly with cancer presence. Since methylation changes can precede diagnosis by several years, cancer patients were matched with controls who remained cancer-free throughout follow-up to minimize bias. Nonetheless, some controls may have developed cancer later, potentially reducing observed group differences. Although this matching strategy mitigated computational bias in the machine learning model, it also reduced the sample size and, consequently, the statistical power.

Although our model was not specifically trained to predict later cancer occurrence, we wanted to explore its performance in this context. The classifier was applied to eight individuals who were diagnosed with cancer more than 12 months after their initial investigation, where no malignancy was initially detected. Interestingly, the two metastatic cases with the longest time to diagnosis were correctly classified, while the remaining six were not detected. This variability likely reflects differences in tumor growth dynamics and the possibility that cancer was not yet present at the time of sampling.

One limitation of this study is the imbalance between the control group and the cancer group. This discrepancy reflects the restricted availability of cancer samples, particularly across tumor subtypes, during the study period. Furthermore, no formal sample size calculation was conducted before study initiation, and the limited number of cases within individual tumor types precluded subtype-specific DMR analyses. Therefore, the identified DMRs reflect general differences between all cancer patients and controls. Future studies with larger, balanced cohorts and predefined sample size calculations are needed to validate these findings, investigate lineage-specific methylation patterns, and strengthen the generalizability of results.

In conclusion, this study demonstrates the clinical feasibility of enzymatic conversion-based library preparation for cfDNA methylation analysis in a low-input setting. We identified significant differences in methylation patterns and DMRs between cancer patients and controls, even within a heterogeneous and clinically relevant cohort. These findings support the potential of methylation as a robust and informative biomarker for cancer detection, maintaining strong discriminatory performance despite substantial biological and clinical variability.

## 4. Materials and Methods

### 4.1. Study Participants

Study participants were recruited between 2018 and 2022 at Örebro University Hospital through the fast-track diagnostic pathway for severe, nonspecific symptoms of cancer employed in Sweden. To enter this diagnostic pathway, patients had to present with one or more of the following symptoms: general malaise, extreme fatigue, reduced appetite, unintentional weight loss >5 kg, prolonged fever, unexplained pain, abnormal laboratory findings (e.g., anemia, elevated alkaline phosphatase, erythrocyte sedimentation rate, or calcium levels), increased health care utilization, increased medication use or a clinician’s intuitive sense (‘gut feeling’) that the patient was seriously ill. All patients underwent a standardized diagnostic work-up, including an expanded panel of biochemical analyses, physical examination, and imaging, such as computed tomography (CT), magnetic resonance imaging (MRI) or ^18^F fluorodeoxyglucose positron emission tomography/CT (^18^F-FDG PET/CT). Diagnostic tissue biopsies were performed when clinically indicated.

All patients entering the cancer pathway were asked to participate. At the time of inclusion, cancer status was unknown. The inclusion criteria for the study were: (1) age 18 years or older (*n* = 236), and (2) availability of EDTA plasma samples (*n* = 232). The exclusion criteria were: (1) a known, ongoing cancer diagnosis at the time of inclusion (*n* = 2), and (2) loss to follow-up, defined as death with a remaining suspicion of cancer without a confirmed diagnosis (*n* = 1). This yielded a final study cohort of 229 participants.

Based on clinical data from medical records, participants were classified into three main groups: (1) those diagnosed with cancer within twelve months of inclusion; (2) those without a cancer diagnosis, who were instead diagnosed with immunologic, infectious, or other nonmalignant conditions, hereafter referred to as controls; and (3) those not diagnosed with cancer within 12 months but later received a cancer diagnosis. These are referred to as Later Cancers. The larger number of controls reflects their greater availability during the study period, whereas cancer patient samples, particularly across the different tumor types represented, were more limited. No formal a priori sample size calculation was performed; instead, we included all eligible samples available during the study period to maximize the robustness of the analysis.

### 4.2. Sample Collection and cfDNA Extraction

Blood samples were collected in EDTA tubes, and plasma was isolated through a double centrifugation protocol: initially at 2000× *g* for 7 min, followed by 16,000× *g* for 10 min. The resulting cell-free plasma was stored at −80 °C until further processing. All samples were frozen within three hours of collection.

cfDNA was isolated from 1.5 to 4 mL of cell-free plasma using the QIAamp Circulating Nucleic Acid Kit (Qiagen, Hilden, Germany). The concentration of cfDNA was quantified using Qubit dsDNA HS Assay Kit (Invitrogen, Waltham, MA, USA) on a Qubit 2.0.

### 4.3. Library Preparation and Sequencing

All samples with measurable cfDNA concentrations after isolation were included in the analysis. Sample processing order was randomized based on cancer status and type, date of cfDNA isolation, and date of sampling.

The cfDNA samples underwent library preparation using the NEBNext^®^ EM-seqTM Kit (New England Biolabs, Ipswich, MA, USA) and the NEBNext Enzymatic Methyl-seq Library Preparation Protocol (Twist Bioscience, San Francisco, CA, USA), with minor modifications.

Mechanical fragmentation was performed only on the control material, which consisted of a pool of hypomethylated lambda DNA (expected methylation <0.5%) and hypermethylated pUC19 DNA (expected methylation 95–98%). Fragmentation was performed using a Bioruptor^®^ Pico sonication device (Diagenode, Seraing, Belgium), with 13 sonication cycles. Each cycle consisted of 30 s on and 30 s off, resulting in final fragment lengths of 240–290 bp.

The library concentration was measured using the same method as for the cfDNA quantification, and fragment length was assessed with a TapeStation 4200 (Agilent Technologies, Santa Clara, CA, USA) using D1000 ScreenTape and reagents.

The Twist Alliance Pancancer Methylation Panel (Twist Bioscience) was used for target enrichment following the Twist Targeted Methylation Sequencing Protocol, with minor modifications. The panel is 1.5 MB in size, covers 126 k CpGs (12 k DMRs), and is designed to cover methylation profiles of 31 cancer types and 47 disease entities based on data from the TCGA database (https://www.cancer.gov/tcga, assessed on 15 October 2025). The hybridization was extended to 16 h, followed by 13 cycles of PCR amplification of the hybridized pools. The concentration and fragment length were assessed using the same methods as described for the libraries.

Sequencing was performed on a NextSeq2000 system (Illumina, San Diego, CA, USA) with 2 × 151 cycles. The sequencing pool consisted of 95% target-enriched libraries, 1% control material library, and 4% PhiX Control v3 Library.

### 4.4. Bioinformatics and Statistics

FASTQ files were generated using BCLConvert (v3.10.12, DRAGEN on-instrument solution, Illumina) and processed through the nf-core/methylseq pipeline (version 2.4.0 and Nextflow version 22.10.6) [41] employing the Bismark Bisulfite Read Mapper and Methylation Caller (v0.24.0) [42]. For methylation conversion controls, the mean CpG methylation was calculated after filtering on 30× depth including their respective full genome, as per instructions from the manufacturer. For samples, a coverage of 100× was used as threshold for calculating the mean CpG methylation level across target regions included in the pan-cancer panel bed file. This threshold led to a mean number of 87 751 covered CpGs in the cohort (SD 17 198 CpGs).

### 4.5. Machine Learning

To reduce statistical errors due to the class imbalance between the cancer group and controls, a subset of samples was analyzed for DMRs. For each cancer patient (*n* = 37), an age- (±2 years) and sex-matched control (*n* = 37) was selected, ensuring that controls remained cancer-free throughout the follow-up period (24–60 months). When multiple perfect matches were available, the control sample closest in sampling date to the corresponding cancer sample was selected. Control samples were sampled blindly for their diagnostic outcome.

Significant DMRs between cancer cases and controls were identified using the wrapped pipeline of DMRichR (v1.7.8) [43], which utilizes dmrseq (v1.24.0) [44] and bbseq (v1.40.0) [45]. For inclusion in the DMR analysis, CpG sites were required to have a minimum coverage of 100× in at least 75% of samples. DMRs were defined as regions containing at least five CpG sites, with all other parameters set to default. Candidate regions were identified based on an adjusted *p*-value < 0.05 for individual CpGs, determined from 74 permutations (one per sample included in the run) and adjusted for sex as a covariate.

Within the DMRichR package, Bsseq was used to generate individual smoothed methylation values, and DMR heatmaps were generated using pheatmap (v1.0.12). DMR annotations and enrichment analyses were obtained and visualized using DMRichCpG and DMRichGenic to determine the CpG locations (islands, shores, shelves, and the open sea) and gene regions (promoters, untranslated regions, exons, introns, downstream, or intergenic regions) for all DMRs, as well as separately for hypo- and hypermethylated DMRs. Fisher’s exact test was used to assess enrichment in DMR locations (CpG and genic context) compared to the background. Additionally, rGREAT (v2.6.0) [46,47,48] was employed for gene ontology analysis based on the genomic coordinate of all DMRs.

Using the average CpG methylation levels in the DMRs identified by DMRichR, a support vector machine (SVM) classifier with a radial basis function kernel was developed, incorporating recursive feature elimination (RFE) for feature selection to distinguish cancer from control samples. Model performance was estimated using five-fold cross-validation. In each fold, the top 20 features were selected via SVM-RFE, and an SVM model was trained and evaluated on the held-out test set.

A final SVM model was trained on the full dataset (*n* = 74) using the top 20 features identified globally. Model performance was evaluated on the same dataset using receiver operating characteristics (ROC) analysis and confusion matrix metrics. Performance was assessed based on area under the curve (AUC), sensitivity, and specificity, both within the internal cross-validation folds and for the final model. A heatmap was generated to visualize methylation patterns of the selected features across all samples.

The final classifier was applied to an external validation set consisting of 144 controls previously unseen by the model. Furthermore, the classifiers’ ability to predict cancer in patients diagnosed after 12 months (Later cancer, *n* = 8) was assessed.

All machine learning analyses were performed in R version 4.4.1 or 4.4.2 using the following packages: sigFeature (v1.22.0) [49] for feature ranking via SVM-RFE, caret (v7.0-1) [50] for model training, cross-validation, and prediction, and pROC (v1.18.5) [51] for ROC analysis and AUC computation.

Additional packages for data handling and visualization included: readr v2.1.5 [52], dplyr v1.1.4 [53], readxl v1.4.3 [54], ggplot2 v3.5.1 [55], and pheatmap v1.0.12 [56].

### 4.6. Statistics

Descriptive statistics for the cohort were performed using IBM SPSS Statistics Viewer (v 29.0) [57]. Shapiro-Wilk tests were conducted to assess the normality of the data. The Mann-Whitney U test and the Kruskal-Wallis test with Bonferroni correction for multiple comparisons were applied to continuous variables. Spearman’s rank correlation and linear regression analyses were used to evaluate correlation and trends.

Figures were generated using R (v4.4.1), or R (v4.4.2, in Rstudio v2024.12.1), and BioRender.com (assessed on 15 October 2025).

## Figures and Tables

**Table 1:** Cohort characteristics divided by controls, cancers and Later cancer diagnoses.

	Controls	Cancer	Later Cancer	Metastasis at dx	*p*-Value
**Sex**					
Female	91 (49.7)	20 (54.1)	3 (33.3)		0.55 ^a^
Male	92 (50.3)	17 (45.9)	6 (66.7)		
**Age (years)**					
Median	71 ^c^	74 ^c^	77		0.001 ^b^
Range	21–91	59–90	62–87		
IQR	59–78	71–83	73–79		
BMI * (kg/m^2^)					0.30 ^b^
Median	24.9	24.8	21.4		
Range	15.0–47.3	15.2–34.6	18.0–29.3		
IQR	21.4–28.1	19.8–27.4	19.7–24.8		
Cancer type ** (*n*)		**37**		**11**	
Carcinoma		25		10	
Haematolymphoid cancers		9		N/A	
CUP		4		1	
**Non-malignant diagnoses (*n*)**	**183**				
Autoimmune disease	23				
Infectious disease	21				
Inflammatory disease	15				
No diagnosis	67				
Other diagnoses ***	57				

* missing values, *n* = 13, ** One patient was diagnosed with two separate tumors: squamous cell carcinoma and cancer with an unknown primary. *** Other diagnoses can be seen in Supplemental Table S1. Overarching categories are indicated in bold. dx = diagnosis, N/A = not applicable. ^a^ Fisher’s exact test, ^b^ Kruskal–Wallis test. ^c^ Dunn’s test indicated a difference between cancer and controls (*p*.adj = 0.0022).

**Table 2:** Model performance in the training cohort. After cross validation, the final model was applied to all samples used in the training, *n* = 74. Sensitivity is presented for the cancer cases in total, and for the different stages. Specificity is presented for the control cases in total, and for the different subgroups.

Disease Stage	Total	TP	FN	Sensitivity%
**Cancer**	**37**	**31**	**6**	**83.8**
Localized	17	11	6	64.7
Metastatic	11	11	0	100.0
Haematolymphoid	9	9	0	100.0
	**Total**	**TP**	**FP**	**Specificity%**
**Controls**	**37**	**31**	**6**	**83.8**
Autoimmune	7	4	3	57.1
Infectious	3	3	0	100.0
Inflammatory	5	4	1	80.0
Other	10	9	1	90.0
No diagnosis	12	11	1	91.7

In bold: overarching categories “cancer” and “control”, with outcomes. Abbreviations: TP = true positive, FN = false negative, FP = false positive.

**Table 3:** Final model performance in the test cohort. The remaining control cases in the cohort were used as an external validation cohort to evaluate the built classifier specificity.

	Total	TP	FP	Specificity%
**Controls**	**144**	**114**	**30**	**79.2**
Autoimmune	16	14	2	87.5
Infectious	18	14	4	77.8
Inflammatory	9	7	2	77.8
Other	45	37	8	82.2
No diagnosis	56	42	14	75.0

In bold: overarching category “control”, with overall outcome. Abbreviations: TP = true positive, FP = false positive.

**Table 4:** Performance of the final model classifier applied to Later cancer cases. The classifier’s ability to predict cancers diagnosed more than 12 months after blood sampling was evaluated in patients who received a cancer diagnosis between 15 and 44 months post-sampling.

Disease Stage	Total	TP	FN	Sensitivity%
**Later cancer**	**8**	**2**	**6**	**25.0**
Localized	4	0	4	0.0
Metastatic	4	2	2	50.0

In bold: overarching category “cancer”, with overall outcome. Abbreviations: TP = true positive, FN = false negatives.

## Data Availability

The datasets supporting the conclusions of this article are included within the article and its additional files. Supplemental Table S1.xlsx contains metadata for all study participants (sex, age, cancer/control, type of cancer, (metastatic, localized, haematolymphoid and differential diagnosis), cfDNA plasma concentration, overall CpG methylation levels. Supplemental Table S2.xlxs contains additional information on the study participants in the group Later Cancers, such as previous diagnosis, time to cancer diagnosis and details on cancer location and disease stage. Supplemental Table S3.xlxs contains a list of all DMRs identified via DMRichR, and whether the DMR was included in the final model or not. The raw sequencing data used and/or analyzed during the current study are available from the corresponding author upon reasonable request, which often requires an ethical permit.

## Supplementary Materials

The following supporting information can be downloaded at: https://www.mdpi.com/article/10.3390/ijms262010165/s1.
