# Supplementary material for: Pan-Cancer Detection Through DNA Methylation Profiling Using Enzymatic Conversion Library Preparation with Targeted Sequencing
Source: Int J Mol Sci. 2025 Oct 19;26(20):10165. doi: 10.3390/ijms262010165 (PMC12564489; doi:10.3390/ijms262010165)
Supplement: Supplementary file 1 [file ijms-26-10165-s001.zip › Supplemental Figure 1. Heatmap Differentially Methylated Regions.docx]

**
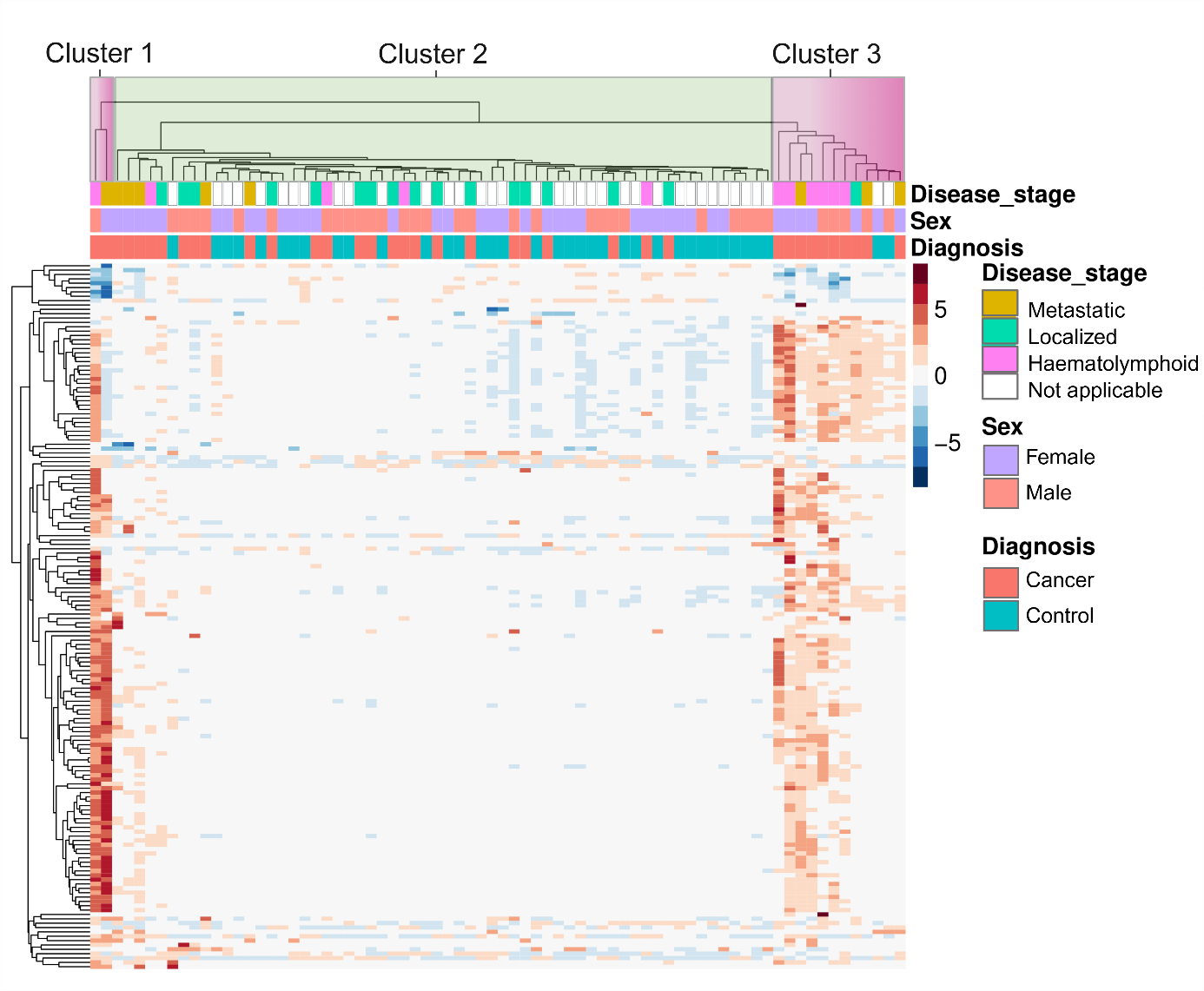
Supplemental Figure 1**. Heatmap from DMRichR pipeline showing the 74 samples clustered based on the z-scores of the 162 significant Differentially Methylated Regions. Samples are labelled based on diagnosis, sex and disease-stage (cancer cases only). Visual interpretation shows three clusters; the first small clusters contains two cancer samples. The large Cluster 2 contain a mixture of cancer and control samples, and Cluster 3 contains 11 samples, all but two cancers. Created in the DMRichR pipeline, modified in BioRender. Adolfsson, E. (2025) https://BioRender.com/9alirjj
