# Supplementary material for: Pan-Cancer Detection Through DNA Methylation Profiling Using Enzymatic Conversion Library Preparation with Targeted Sequencing
Source: Int J Mol Sci. 2025 Oct 19;26(20):10165. doi: 10.3390/ijms262010165 (PMC12564489; doi:10.3390/ijms262010165)
Supplement: Supplementary file 1 [file ijms-26-10165-s001.zip › Supplemental Figure 2. Exemplary plot of a Differentially Methylated Region.docx]

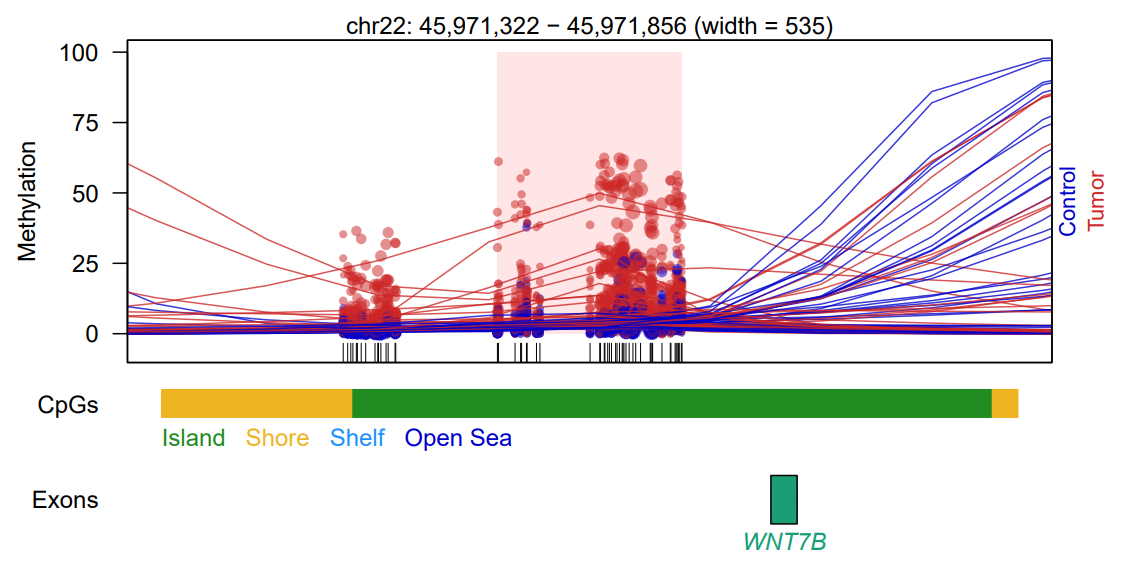


**Supplemental Figure 2**. One of the DMRs selected for the final classifier model. The DMR is in close proximity to *WNT7B*. Compared with control samples (blue), cancer samples (red) presented with hypermethylation, with an overall methylation difference of 6%. The DMR is 535 bp long and contains 42 CpGs.
